# Development of electronic medical records for clinical and research purposes: the breast cancer module using an implementation framework in a middle income country- Malaysia

**DOI:** 10.1186/s12859-018-2406-9

**Published:** 2019-02-04

**Authors:** Nurul Aqilah Mohd Nor, Nur Aishah Taib, Marniza Saad, Hana Salwani Zaini, Zahir Ahmad, Yamin Ahmad, Sarinder Kaur Dhillon

**Affiliations:** 1 Data Science and Bioinformatics Laboratory, Institute of Biological Sciences, Faculty of Science, University of Malaya, 50603 Kuala Lumpur, Malaysia; 2 Department of Surgery, University Malaya Medical Centre, 50603 Kuala Lumpur, Malaysia; 3 Department of Oncology, University Malaya Medical Centre, 50603 Kuala Lumpur, Malaysia; 4 Department of Information Technology, University Malaya Medical Centre, 50603 Kuala Lumpur, Malaysia

**Keywords:** Electronic medical record, Breast Cancer, Database mirroring, Medical system, Quality implementation framework

## Abstract

**Background:**

Advances in medical domain has led to an increase of clinical data production which offers enhancement opportunities for clinical research sector. In this paper, we propose to expand the scope of Electronic Medical Records in the University Malaya Medical Center (UMMC) using different techniques in establishing interoperability functions between multiple clinical departments involving diagnosis, screening and treatment of breast cancer and building automatic systems for clinical audits as well as for potential data mining to enhance clinical breast cancer research in the future.

**Results:**

Quality Implementation Framework (QIF) was adopted to develop the breast cancer module as part of the in-house EMR system used at UMMC, called i-Pesakit©. The completion of the i-Pesakit© Breast Cancer Module requires management of clinical data electronically, integration of clinical data from multiple internal clinical departments towards setting up of a research focused patient data governance model. The 14 QIF steps were performed in four main phases involved in this study which are (i) initial considerations regarding host setting, (ii) creating structure for implementation, (iii) ongoing structure once implementation begins, and (iv) improving future applications. The architectural framework of the module incorporates both clinical and research needs that comply to the Personal Data Protection Act.

**Conclusion:**

The completion of the UMMC i-Pesakit© Breast Cancer Module required populating EMR including management of clinical data access, establishing information technology and research focused governance model and integrating clinical data from multiple internal clinical departments. This multidisciplinary collaboration has enhanced the quality of data capture in clinical service, benefited hospital data monitoring, quality assurance, audit reporting and research data management, as well as a framework for implementing a responsive EMR for a clinical and research organization in a typical middle-income country setting. Future applications include establishing integration with external organization such as the National Registration Department for mortality data, reporting of institutional data for national cancer registry as well as data mining for clinical research. We believe that integration of multiple clinical visit data sources provides a more comprehensive, accurate and real-time update of clinical data to be used for epidemiological studies and audits.

## Background

In 2012, Globocan reported that Malaysia had the highest breast cancer mortality in the Southeast Asian Region based on estimation from neighboring countries and regional registries in Malaysia [1]. University Malaya Medical Centre (UMMC) Surgical Breast Unit has produced the first breast cancer outcomes data in Malaysia [2–4]. The institutional survival rates differ tremendously with a published population based study but further details on stage at presentation and other clinical variables were not available for nationwide outcome analysis [5]. In Malaysia, data capture methods had been manual and done retrospectively by tracing notes of patients’ clinical characteristics and treatment characteristics. This method is expensive with high probability of missing values and inaccuracies. Reducing manual work by automated data capture systems has been cost effective especially in the light of increasing burden of salary costs to hospitals. In a typical clinical set up, these primary data are used for surgical audits in measuring the hospital performance, while the secondary use data will be used in epidemiological analysis in breast cancer outcome research.

A typical breast cancer patient’s journey through diagnosis and treatment involves multiple disciplines and departments. Breast cancer diagnostics require input by surgical, radiological and pathological disciplines. In such circumstances, efficient data management and computational workflows are needed to generate meaningful clinical data, rather than having textual data and building algorithms to mine retrospective data. With the increasing use of EMR data in research, EMR has high potential in becoming a major data source for future medical research and clinical service evaluation of a practice [6–8]. The rapid increase in quantity of clinical information in electronic format makes secondary use of clinical data a candidate for big data solutions [9, 10]. The availability of data extraction techniques in the data repository opens up more avenues in addressing research questions [6, 11, 12]. Prospectively managed data would provide clean data and more accurate data leveraging the power of Artificial Intelligence to detect and uncover clinical relationships and knowledge [13]. Aggregating data from different sources in healthcare and research is important [14] to discover hidden knowledge from different sources in healthcare [15]. The effectiveness and data quality of records can be improved through the enhancement of the clinical research database features. Elements needed for a successful clinical research database include engagement of clinicians, utility for research and the ability to integrate with the legacy systems [16, 17].

The revolutions caused by advanced computing power, advanced informatics and communication technology have changed the way clinical data are stored and used. Today almost every hospital realizes the need of storing its clinical data sets electronically in order to increase the quality of healthcare service and data. Many countries have embarked into the management of huge amount of clinical data using Electronic Medical Records systems. Examples are National Electronic Health Records (NEHR) in Singapore [18], National Programme for Information Technology (NPfIT) NHS Care Records Service in the United Kingdom [19], The Royal Children’s Hospital Electronic Medical Record in Melbourne [20], Allscripts, eClinicalWorks [21], EPIC [22], McKesson, Care 360, Cerner, OPTUM Insight, NextGen, and Greenway in the USA [22–26]. Malaysia, although being in the forefront in providing one of the best medical care in the world, is still at a very immature stage concerning clinical data management. Electronic Medical Record (EMR) and Hospital Information Management System (HIMS) in Malaysia is still in the preliminary stage [27].

In line with the Vision for Health Statement [28–32], Malaysia has initiated effort in providing a platform for the country’s transition in promoting information technology usage in health sector during the Eighth Malaysia Plan (2000–2005) [28, 33]. The Ninth Malaysia Plan (2006–2010) [29] highlighted on strengthening the Health Information System, to improve the point-of-care service and patients’ health information access. To facilitate this, a nationwide information system is introduced by focusing on enhancing digital information structure expansion. Tenth Malaysia Plan aimed to achieve integration and interoperability between various hospital information systems (HIS) [30, 34]. It has been an ongoing effort in improving the clinical data management system as being mentioned in The Eleventh Malaysia Plan [32], Malaysian Health Reference Data Model [35], and Malaysia Health Data Warehouse [36].

During the implementation, it was found that there were inadequate integrated planning of HIS where different hospital uses individual stand-alone systems, lack of uniformity in operational policies of the hospitals and weak governance in securing the privacy of clinical data [31]. However till date, the success rate has been low. There is an absolute urgency in developing a reliable, integrated and interoperable Health Information Management using an implementation framework [37]. In this paper we present our Breast Cancer Module in the University Malaya Medical Center EMR which is developed using the Quality Implementation Framework (QIF) [38]. The QIF is an implementation framework used to introduce new services or workflows in a healthcare setting. This paper highlights the challenges faced especially in a developing country setting with limited resources and funds, along with the development of the system using an infrastructure that matches the hospital environment.

## Methods

**Fig. 1:**
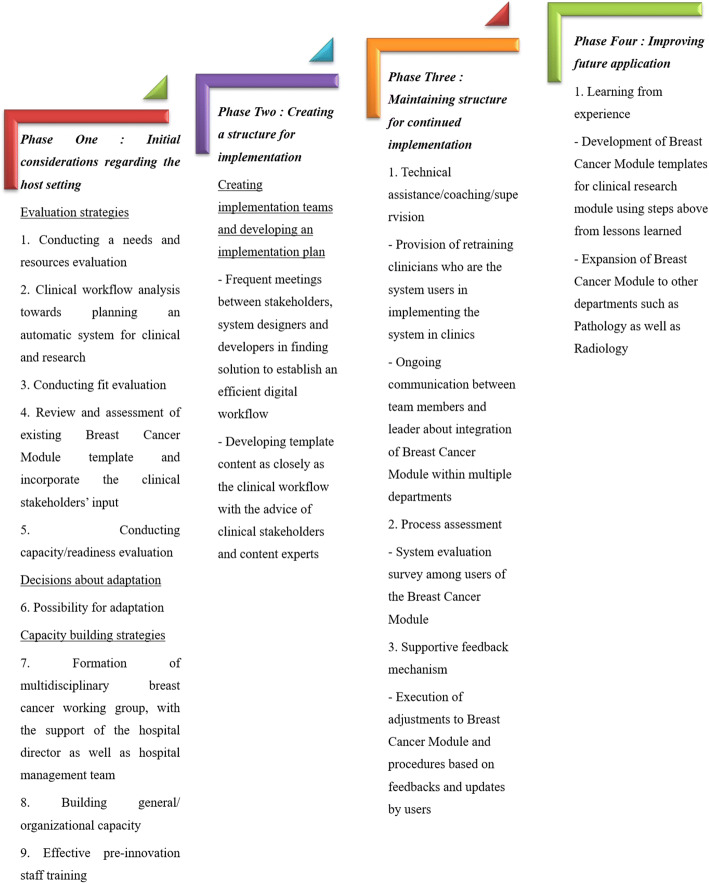
Establishing i-Pesakit© Breast Cancer Module at the University Malaya Medical Centre using the Quality Implementation Framework

The Quality Implementation Framework (QIF) is adopted because it synthesizes existing models and research support to provide a conceptual overview of the critical steps that comprise quality implementation [38]. The QIF contains four temporal phases and 14 distinct steps as described in Fig. 1.

**Fig. 2:**
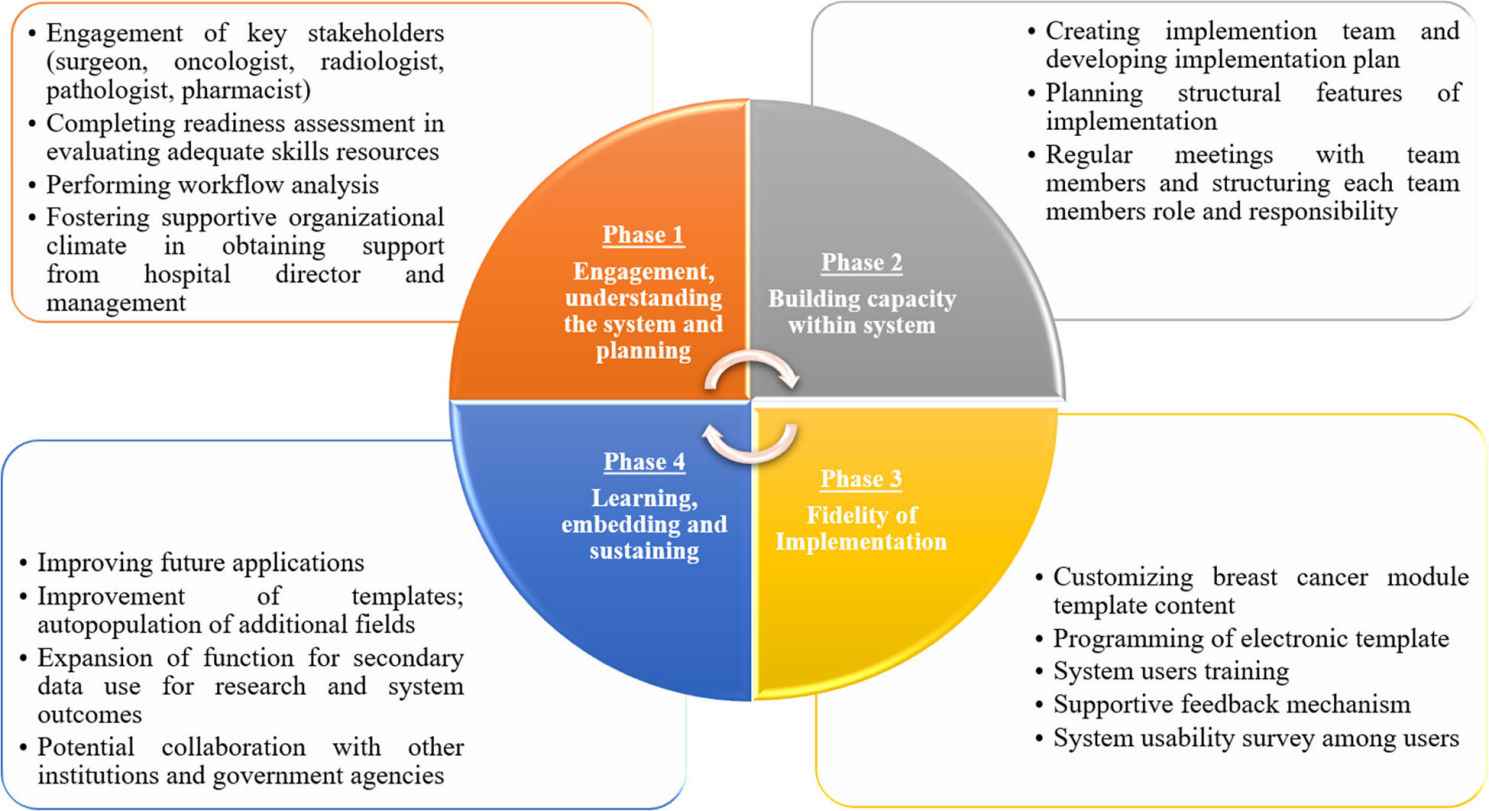
Framework and development of i-Pesakit© Breast Cancer Module adopted from Quality Implementation Framework (QIF) Model

These steps comprise four QIF phases: (i) initial considerations regarding the host setting, (ii) creating a structure for implementation, (iii) ongoing structure once implementation begins, and (iv) improving future applications. In our study, we have completed to Phase 4, providing steps to improve future applications. Summary of the four implementation phases and 14 critical steps in the Quality Implementation Framework [38] that are associated with quality implementation is presented in Fig. 2.

## Phase one: Initial considerations regarding the host setting

### Conducting a needs and resources evaluation

At the initial stage, a group comprising a multidisciplinary team of expertise was formed to evaluate different areas of assessment, in terms of needs, resources and requirements. The breast cancer module is by itself a multidisciplinary clinical practice encompassing Surgery, Oncology, Radiology, Pathology and Pharmacy departments. The stakeholders and content experts are clinicians who are actively involved in day to day clinics from diagnosis to treatment and follow-ups, while the structure of system is designed by Bioinformaticians and developed by Information Technology (IT) experts. More importantly, the main stakeholders which are the Hospital Management Board, Ethics Committee and Patient Information Department responsible for policy making on clinical data in regards to patient confidentiality were engaged. Before venturing into designing a new model for the breast cancer clinical and research data reporting, an assessment was conducted to identify specific issues and concerns in the current practice at the hospital. We identified concerns on data privacy and confidentiality under the Malaysia Personal Data Protection Act (PDPA) 2010 [39]. Hence, several discussions to develop a system model and governance that is compliant to both the PDPA and research processes were done with these committees.

### Compliance to personal data privacy and confidentiality issues

Personal Data Protection Act 2010 (PDPA) compliance [39]; a set of regulations that provides data privacy and security provisions for protecting clinical information was discussed with the UMMC Medical Records Department which is bound by the Malaysia Health Care Act and the National Archive of Malaysia Act. Data usability for research was conducted through safe and secure use of technology to automate data transfer into the UMMC Clinical Research Knowledgebase via de-identification of primary patient records. This prototype fulfils the Health Level-7 standard (HL7), an international standard for data transfer of clinical and administrative information. The details of the prototype are illustrated in the Results section. In the case of using clinical data with identifiers, we had to obtain written permissions from the ethics committee for a given duration required for the job execution.

### Needs on national cancer registry reporting

The importance of a national cancer registry lies in the fact that they consolidate accurate and complete clinical cancer data as cancer control and epidemiological research, public health program planning, and patient care improvement. Ultimately, a complete national-level system of cancer registry can assist clinicians and researchers in understanding cancer better and maximize our resources to the best outcomes in treatment and prevention.

### Assessment of breast cancer reporting

The UMMC Breast Cancer Registry begun in 1993 with data amounting to over 6000 individual patient data. This single page proforma that was consolidated into a spreadsheet had essential data that enabled UMMC to be the first to publish breast cancer outcomes in Malaysia and had enabled collaboration internationally to establish outcomes in Southeast Asia and Asia [2–4]. A more complex UMMC Breast Cancer Registry Clinical Proforma was developed in 2009, which included details on diagnosis and treatments and other clinical characteristics involved in risk and prognosis of breast cancer patients. Data were collected manually through patients’ visits through diagnosis and treatments prospectively and retrospectively from medical records. The work process was labor intensive and required training of non-medical personnel. Other manual workflow limitation includes the unavailability of keeping track of patients’ status, including recurrence and survival status.

### Conducting a fit assessment

UMMC Electronic Medical Records (EMR) i-Pesakit© system was developed in January 2012 by the UMMC Department of Information Technology with seven main modules to cater to patient management activities which include patient registration, outpatient, inpatient, emergency medicine visits, billing, folder tracking and reporting. The system has been operational since 1st July 2012. The system was further developed to cater to medical records requirements, which include clinical documents, orders and results. The expansion of UMMC EMR project started in September 2013 and was implemented as a pilot study in the staff health clinic a year later. The pilot project was extended to Primary Care Medicine clinic and the Breast Unit, and later Department of Surgery. The system is registered under the copyright act in July 2016 with Intellectual Property Corporation of Malaysia (MYIPO) and has already been commercialized. From time to time, various iterative improvements had been made to the system to be able to work as required by clinicians. To date 99% of UMMC clinical areas implement the i-Pesakit© EMR.

However, the current i-Pesakit© system, only covers generic patient data which could only generate basic audit reports, which is not aligned to the bigger aspiration of data mining for research outputs. Challenges in providing manual data transcription by salaried personnel provided avenues of building cost effective solutions for data management such as audits and for research. The primary objective was to collate accurate clinical data encompassing risk and prognostic variable and ensuring the ability to integrate with the legacy system. Hence, the assessment of institutional data management and users were aligned to build new solutions, tied up with the current hospital’s EMR system which fits the environment and has been used for 6 years.

### Conducting a capacity/readiness assessment and decisions about adaptation

Since EMR was implemented in 2012, the loss and misplacement of patient records and x-ray films, originally in physical paper folders were drastically alleviated. Ultimately, an ideal hospital information system should allow seamless connections and integration of other clinical departments to improve clinicians’ work performance and produce positive healthcare institutional outcomes.

Readiness for adaptation was evident as the department of surgery was slotted for complete breast cancer surgery department prototype module usage in 2016 which was designed and developed from scratch by the critical stakeholders.

In filling out the research data management gaps within this research hospital, the status of EMR implementation process and responses of clinicians on its impact on their routine in patient care has been positive. This allows the establishment of ground work for next phase of breast cancer research module.

## Decisions about adaptation

### Possibility for adaptation: Development of the i-Pesakit© Breast Cancer Module

In order to improve the efficiency of clinical data management system in i-Pesakit©, restructuring of information-capture process and upgrading the system flow through engagement with the doctors’ clinical work processes are crucial. A mechanism to translate paper-based operations to digital data capture is introduced and entries are reflected in the hospital’s EMR system, which further extends to clinical audits based on primary data obtained. Uptake of EMR use in Breast Unit was largely due to the age cohort of the users, which are mainly Master of Surgery surgical trainees and medical officers (25 to 35 years old) who are quick to adapt to migration of work routine to a digital clinical workflow.

Due to poor retainment of staff in this public hospital, it is hence practical to move towards digital platforms. However, senior clinicians may perceive the system as hindrance to effective clinical work due to the inconvenience and concentration issues in multitasking between typing, paying attention to the computer screen rather than developing a rapport with the patients. The impact of the system implementation contributes to both service and clinical data quality, as well as job performance.

### I-Pesakit© Breast Cancer Module construction and content

Since the breast cancer workflow encompasses different departments in the hospital, it is ideal to design an interoperable system for sharing records between departments as well as structuring an availability of standards for integration of various clinical workflows.

Engagement of critical stakeholders from these units were agreeable to a workflow of interoperable system that would be adaptable to practices in these units. The framework and development of i-Pesakit© Breast Cancer Module, adopted from the QIF model is demonstrated in Fig. 2.

The source materials used in this study were obtained from the UMMC. Data sources include case report forms (CRF), manual legacy Excel sheets, in house EMR system used by the clinicians, clinical workflow requirements gathered from the users such as clinicians and nurses. The i-Pesakit© Breast Cancer Module is based on the UMMC Breast Cancer Registry Clinical Proforma, which include details on diagnosis and treatments and other clinical characteristics involved in risk and prognosis of breast cancer patients.

### System design and construction: Mapping out clinical workflows

The flow of patient registration, diagnosis and treatment workflows was studied carefully. This was the key in making decisions on the development of the breast cancer module. Standardized data entry forms that are compatible with the EMR system were created, by translating these paper-format case report forms into an electronic case report form (e-CRF) before embedding the e-CRF design into EMR.

**Fig. 3:**
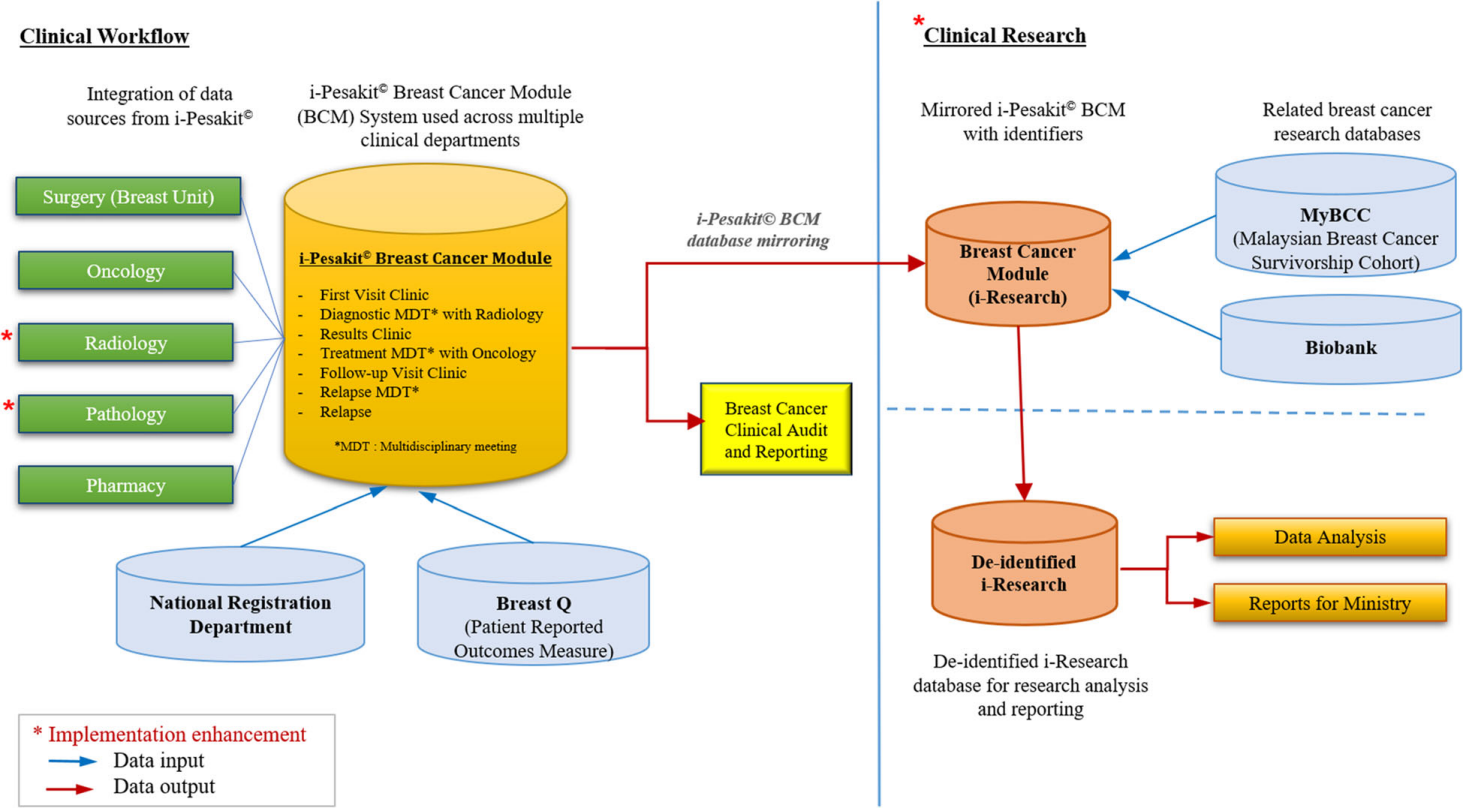
Architecture system of the UMMC i-Pesakit© Breast Cancer Module

The system workflow is illustrated in Fig. 3, where the design of breast cancer module in EMR consolidates data input from the Surgery and Oncology departments and is merged with the existing patient details in EMR. To date, we have successfully developed a prototype module which includes Surgical and Oncology, as well as the Pharmacy departments. Future works will be done in integrating other clinical departments such as Radiology and Pathology to enhance the system interoperability and increases data accuracy. The i-Pesakit© Breast Cancer Module is used widely in clinics, wards, as well as during diagnostic and treatment plan multidisciplinary meetings with Radiology and Oncology departments in discussing diagnosis and treatment plans.

**Fig. 4:**
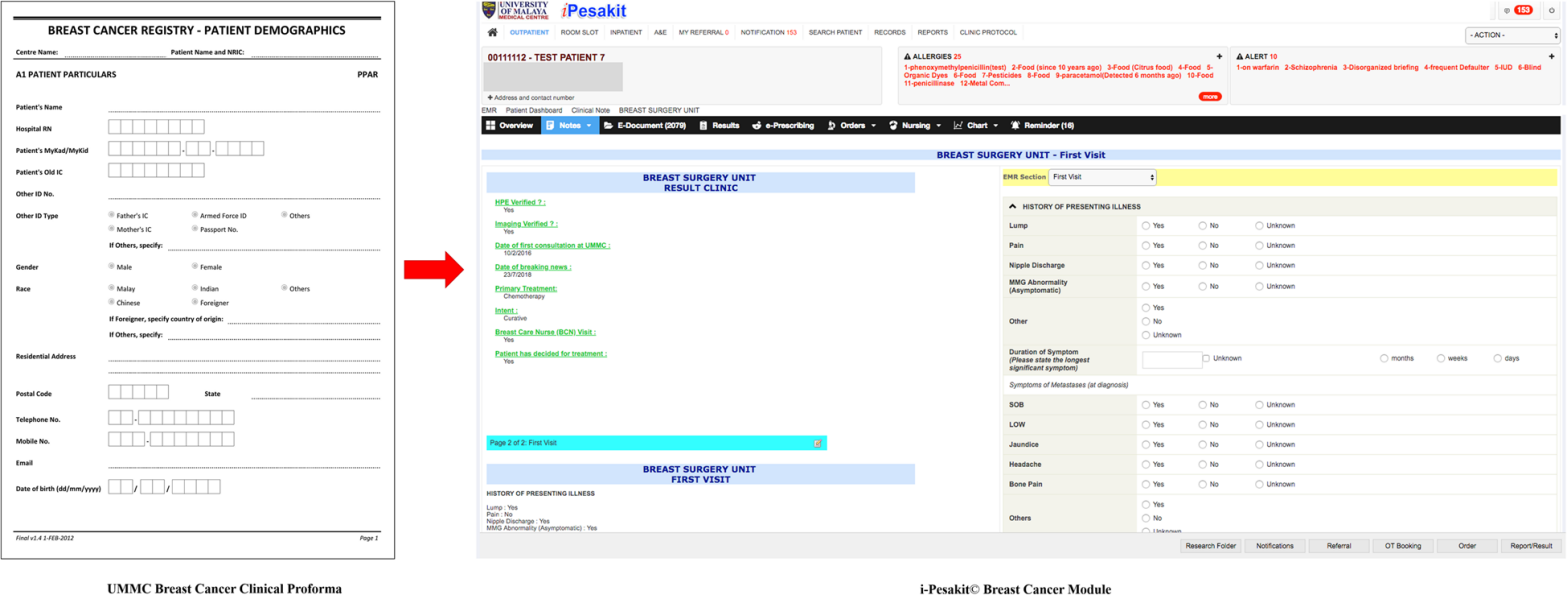
Translation of breast cancer proforma into web-based UMMC i-Pesakit© Breast Cancer Module

The construction of the breast cancer module involves studying the requirements of the organization. Based on the workflow, a prototype was built by translating the CRF into computerized web forms, linking with the existing rudimentary patient details available in the EMR. The translation of the CRF into a web-based i-Pesakit© Breast Cancer Module is shown in Fig. 4.

A total of 42 case report forms (CRFs) were translated during the process of developing the breast cancer module; represented by 9 categories which are First Visit, Diagnostic Multidisciplinary Team (MDT) with Radiology, Results Clinic, Admission for Surgery, Treatment MDT with Oncology, Oncology Visits, Oncology Treatment Summary and Relapse.

The EMR was implemented on MariaDB and open source systems for the system developmental work. Currently, the breast cancer module contains data sources from the Surgery, Oncology and Pharmacy departments. The fully automated EMR breast cancer module is being used in the clinical visits as well as for audits, while further enhancement will be made in integrating other related sources from Radiology and Pathology for a more compact system.

### Obtaining explicit buy-in from critical stakeholders and fostering a supportive community/organizational climate

The hospital management board, inclusive of hospital director, Patient Records Department, Hospital Informatics Department were among the crucial stakeholders with decision making power were engaged very early in the project. The vision for the hospital to apply the 4th Industrial Revolution [37] was very much aligned to this project. Hence, the support received to further this project from critical stakeholders was very important in the development process. The second step was through engagement of content experts, clinicians include surgeons, oncologists, radiologists, pathologists, and pharmacists, as key stakeholders in each of these departments. The engagement was done in stages, where surgical and oncology departments as well as e-prescription of chemotherapy with the pharmacy department were engaged for the pilot project.

The collaboration between academics, graduate students and programmers was able to foster close relationships, sharing of tasks despite shortage of manpower within the service sector. The researchers played a role in providing detailed logs of changes and became the conduit between the user and the programmers. The greatest disincentive if we are not able to produce an automated system is challenges to salary personnel to continue manual data collection.

### Building general/organizational capacity

Organizational capacity includes increasing more system designers and developers, and task sharing between academia, graduate students and project-based programmers. We discovered organizational policies with regards to developing IT solutions for handling of digital data in the confines of the PDPA needs improvement and proper policy and protocols in place to ensure smooth implementation.

This includes processes of obtaining permission for students and researchers to work within the hospital departments where initial challenges were encountered and resolved when trust and clear boundaries were defined.

### Staff recruitment/maintenance

The implementers of the system are the clinicians of UMMC, so training and ongoing support will be given to users to build their capacity in knowledge about the system. A breast care nurse is assigned to oversee this day-to-day system use in the clinic and holds the role as a middle person between the implementers and system designers to provide feedbacks about the system.

**Fig. 5:**
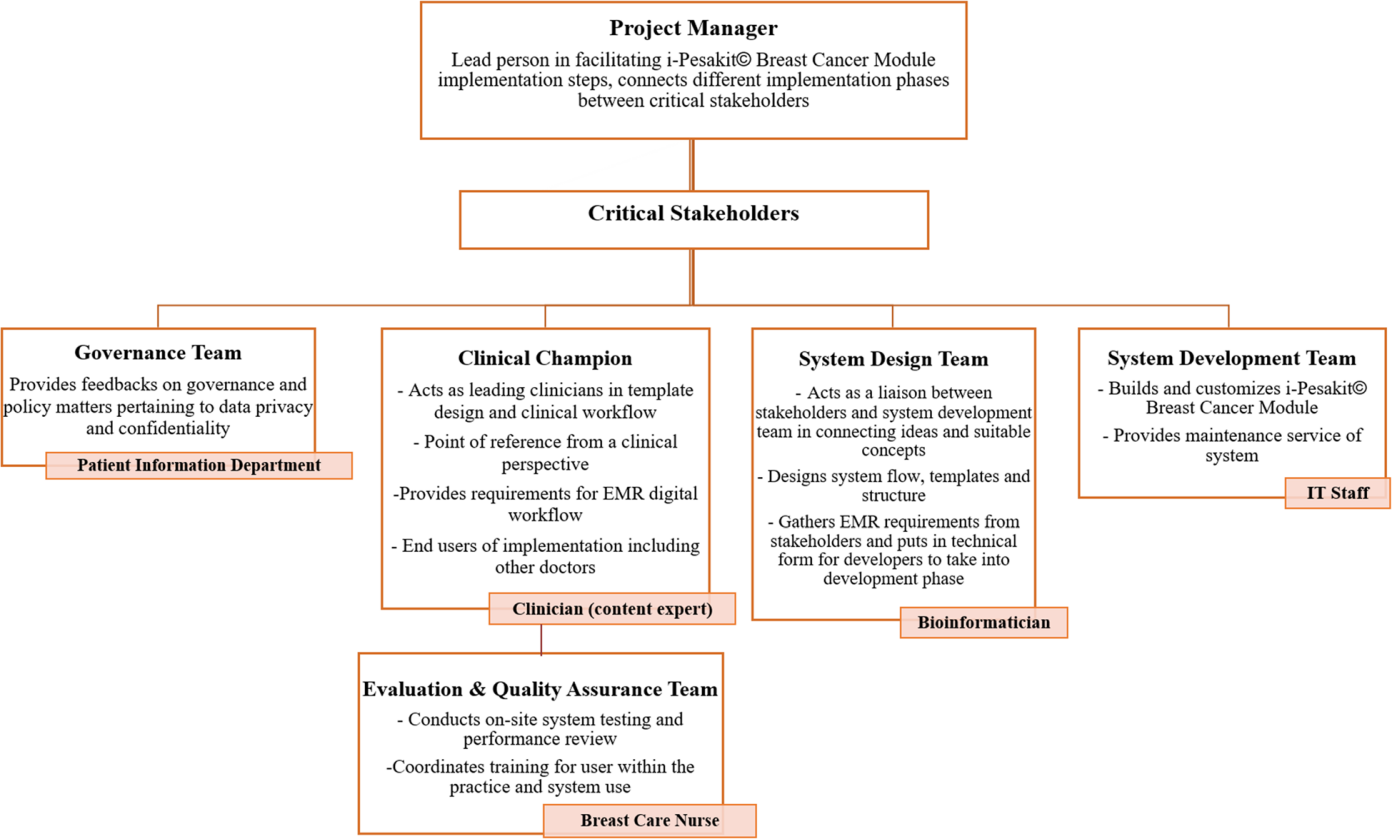
Crucial members of i-Pesakit© Breast Cancer Module implementation team

Figure 5 describes the team members involved directly or indirectly in the project from permissions to execution and testing. There are different categories of roles in the implementation team involving the project manager who is the quarterback of the EMR implementation group, project team members include critical key stakeholders; the hospital management, governance team, physician champions, bioinformaticians and IT staffs in designing and building the EMR module, as well as nurse leads for evaluation and quality assurance team in doing on-site testing and user trainings.

### Effective pre-innovation staff training

A protocolled training session is carried out for new rotating medical officers in the unit every 3 months. This training will be conducted by the breast care nurses.

## Phase two: Creating a structure for implementation

In order to ensure the success of any implementation, the people involved need to have the right expertise and roles and secondly a viable plan ahead of the development. In this project, our team is multidisciplinary with distinguished roles who have been assigned with dedicated tasks and timelines. A summary of the team members with job scope is presented in Fig. 5.

### Creating implementation teams

There are five groups of crucial members in this EMR implementation; (i) project manager and critical stakeholders include (ii) hospital management and governance team, (iii) physician champions, (iv) system design and development, as well as the (v) evaluation and quality assurance teams. The project manager is the lead person in facilitating these implementation steps, connects different implementation phases and coordinate the planning, design, development, and testing phases between team members. The hospital management and governance team from the Patient Information Department provide feedback on governance and policy matters pertaining to data sharing, privacy and confidentiality. Physician champions have credibility with clinical staffs and hospital administration, to promote value of the innovation through stakeholders engagements. They are also the main point of reference from the clinical perspective, also as content experts and EMR functionalities so the digital workflow matches closely to the actual clinical workflow. The system designers (bioinformaticians) act as a liaison between physician champions and system development team (IT staffs) in connecting ideas and suitable concepts. Bioinformaticians design the digital system workflow, templates and structure through gathering EMR requirements from physician champions and put in technical form for system developers to take into development phase. IT staffs are responsible in building, customizing and deploying the breast cancer module, as well as providing maintenance service of the system to be conducted by the evaluation and quality assurance team (breast care nurse). Nurses conduct on-site system testing and performance review and coordinates training for users within the practice and system use.

Workflow analysis is done in the planning stage where bioinformaticians study the existing clinical work processes, looking for opportunities for improved efficiency, assessing and designing new workflows and system structure and developing a transition plan towards a digital clinical workflow environment. Good communication is crucial between bioinformaticians and clinicians, in coming up with the best solution of improvised workflow that is time effective and user friendly for the frontline implementers.

The IT experts are responsible in deploying and constructing the EMR system. Participation of clinical staff in the implementation process increases support for and acceptance of the EMR Breast Cancer Module implementation. In line with the hospital management support and participation of clinical and non-clinical staff, having an interdisciplinary implementation group involves direct stakeholders working together, where a better EMR system can be delivered faster and with less problems.

We foresee difficulties in implementation and monitoring of the busy medical officers hence, qualified staff on-site to oversee and support implementation were played by breast care nurses. As a central role on the team, they understand these EMR clinical workflows, inspire clinical staff to embrace change, and drive consensus among other clinical staff. There is a close collaboration and feedback mechanism between implementers, supportive team (breast care nurses), physician champions, as well as EMR design and development team in fine tuning the system from time to time.

### Developing an implementation plan

Implementation plan involved designing the pilot system and going live with support and specific tasks starting with First Visit template (Fig. 4) for all new cases, as progressively include follow-up for cancer patients. There is a mechanism in place to produce quality entries as medical officers are accountable to document EMR professionally, to ensure the clinical service as well as data are high quality.

Progressively other templates were used, through the development of e-Prescription of chemotherapy from the Oncology department to Pharmacy department, as well as specific clinical templates for the departments of Radiology and Pathology.

Eight months after the implementation process began, the prototype system went live on February 2016. Support for users is provided with a two-tiered approach. On-site support is available from the trained breast care nurses who understands the EMR workflow to oversee the system. If the problem still persists, an information technology services staff member is called for support by phone. This has allowed the majority of technical problems to be solved locally.

In the first few months after implementation, occasional meetings between clinicians and bioinformaticians were called to address specific issues that arose. Implementing an EMR breast cancer module system is challenging. It requires good planning, strong physician leadership and supportive clinical and non-clinical staff. The most immediate benefits of the EMR breast cancer module system have been accurate diagnostics, treatment plans, legible notes and prescriptions, and lower transcription costs.

### Breast cancer module within the electronic medical record

The focus of the study was extended to integrating and enhancing the system interoperability according to clinicians-specific function requirements, support and maintenance (impact on technical architecture), as well as data availability and sharing amongst clinicians in providing meaningful representation of patient data electronically. In the testing stage, further enhancement effort was done to improve user friendliness, according to accurate clinical and nursing work flows.

However, the current EMR system does not provide a strong basis for clinical research, as the data structure is scattered and not standardized. The mortality data from the National Registration Department will soon be linked to the EMR, which is useful in survivorship analysis research.

### Clinical research database: The database mirroring concept

A critical factor for successful utilization of available EMR clinical data for research is the access, management and analysis of integrated patient data, within and across different functional domains. For example, most clinical and basic research data are currently stored in disparate and separate systems, and it is often difficult for clinicians and researchers to access and share these data. Equally important is the assurance within EMR systems of security, with confidentiality, integrity and general trustworthiness to meet the requirements for high quality research data.

In innovating a practical approach to develop a clinical research workflow and framework, the EMR System Mirroring was designed to provide an economical solution for rapid, reliable, robust, automatic failover between two database systems, making mirroring the ideal solution in minimizing redundant components and risk of human error transcriptions.

The clinical research database use and workflow is in line with the Clinical Data Interchange Standards Consortium (CDISC) [40, 41] which supports the electronic acquisition, exchange, regulatory submission and subsequent archiving of clinical research data. Developing a new system for clinical research is not practical due to overhead cost of programming, requirements development, designing and infrastructure, hence EMR mirroring for the purpose of clinical research is the best and cost-effective solution. Successful case studies have proposed to automate data transfer from primary EMR models for clinical research [42–44] without using vendor specific third party clinical research databases. Hence, in this paper we propose to use the primary EMR to be mirrored and de-identified for research purposes. The mirroring could be done using a middleware to facilitate data transfer.

Quality assurance mechanisms are needed to ensure that the EMR system adheres to certain quality characteristics. The governance framework and design structure of EMR fulfils the Malaysian Medical Council Confidentiality 2011 Guideline [45], which supports clinical data usage for research, clinical audit and secondary use.

## Phase three: Ongoing structure once implementation begins

### Ongoing implementation support strategies

#### Technical assistance/coaching/supervision

Training clinicians and nurses to effectively use the breast cancer module into their clinical workflow is an important step to a successful implementation in making sure data is entered in a standardized manner. Quality, safety, and integrity of data are protected, while it increases the efficiency of clinical care, especially through point of care (POC) adoption in the clinical setting. Ongoing training will be conducted from time to time when there are additional functionalities introduced to the system, including producing clinical audits and contribution to national statistics in measuring the hospital’s performance.

The challenging environment of staff shortage affects the time taken in updating the systems according to clinical needs. There is a need of programmers to resolve feedback quickly as not to lose momentum and affecting the digital clinical workflow. Training of medical officers by breast care nurses, briefing for each new staff to the team is important to ensure the EMR breast cancer module usage is maximized. The breast care nurses’ expertise has knowledge on how daily operations work in a clinical setting, so testing and quality reviews can be performed for data security, proper functionality within the department, performance review, and to verify the system closely matches the actual clinical workflow.

The common barriers in implementing this new system are users’ resistance to use the digital template and wrongly using template usage. This is solved by creating an EMR workflow which matches the clinical workflow closely to ease the transition of manual to digital clinical workflow among clinicians.

The information technology lead is responsible for deployment and operation of the software and hardware such as workstations, in providing IT support in servers and connection issues. Constant change and improvement of system is conducted from time to time in improving the system usability and performance.

### Process evaluation

#### System testing and evaluation

Initial adaptation of the breast cancer surgery department module was also tested with the users in the department. The pilot system has gone live since February 2016, and a usability testing survey was conducted to test the readiness of EMR adaptation in the breast cancer workflow. The system test evaluation survey material [46] was adapted from Evaluation of Electronic Medical Records Questionnaire [47].

**Fig. 6:**
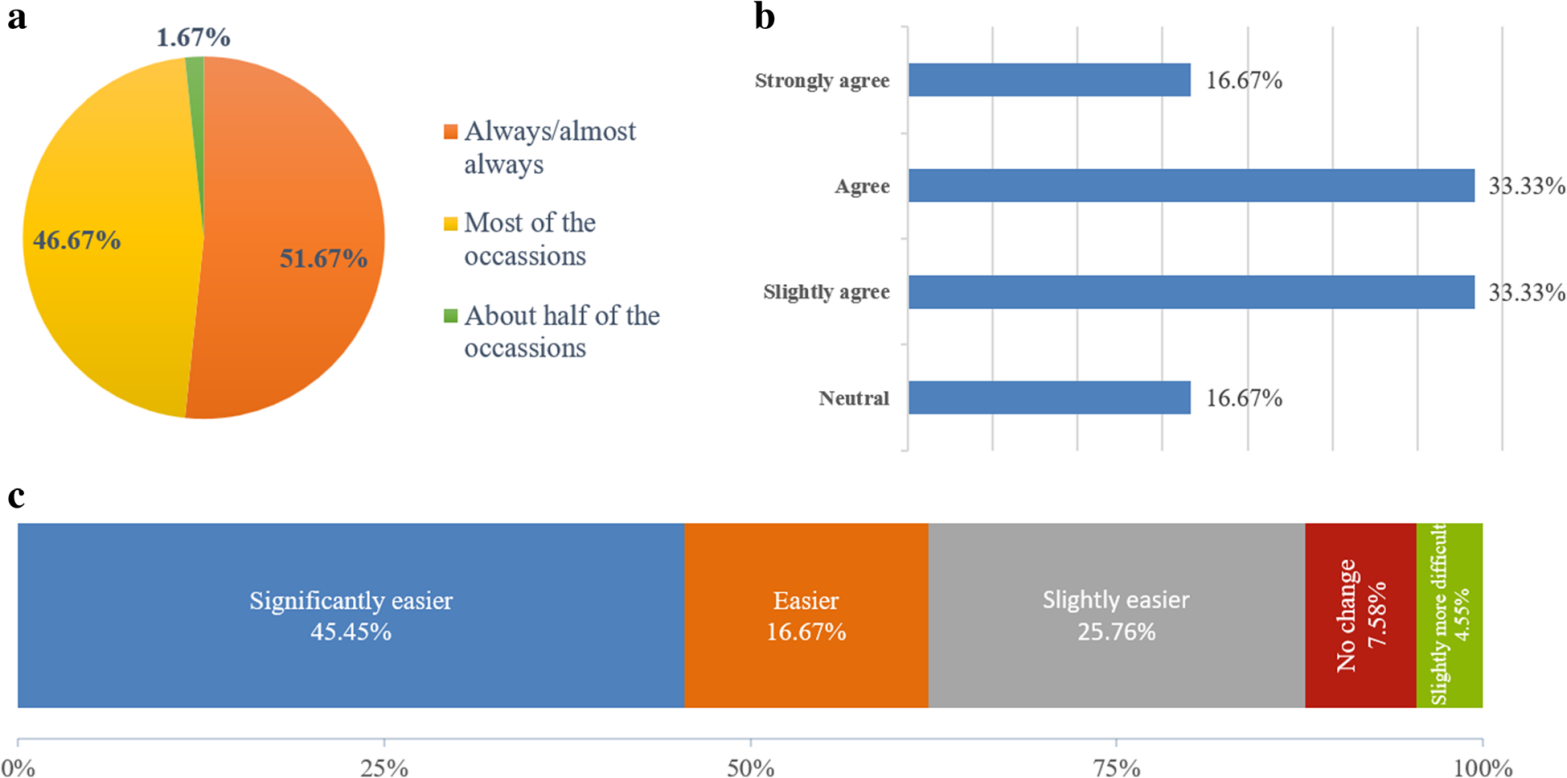
Usability survey on clinicians’ perceptions of i-Pesakit© BCM (**a**) Usage of system in clinical work (**b**) Clinicians’ acceptance in finding this system worth the time and effort to be used (**c**) Effect on clinical workflow by using the system

Following the system usability amongst the department of surgery users, it was found that the clinicians welcomed migration to a new routine from paper-based clinical notes into a fully digitized environment. Overall, the response was good in terms of using the EMR which was piloted in the Surgery department (Fig. 6), hence the climate for up taking EMR for breast cancer was positive.

During the first year of i-Pesakit© Breast Cancer Module implementation in the Breast Unit of Surgical Department, a system usability survey was conducted to measure clinician’s perceptions of the recently implemented system in determining their satisfaction level of using the system in their daily clinical routines (Fig. 6a, b, c).

Positive feedbacks were given by clinicians, where 98.34% of them use the system frequently in their clinical work. The effect of using the breast cancer module system has made their workflow smoother as well as easier, while 7.58% of them experience no change in convenience and 4.55% find the system complicated. However, 83.33% of clinicians agree that the breast cancer module is worth the time and effort to be used. More user training is required, to familiarize them with the system in order to achieve maximum benefit of the system, especially in reducing time taken in documenting patients’ clinical data.

From time to time adjustments are made by the programmers based on feedbacks given by users. However, in order to expedite the process of correcting bugs or errors, more programmers can be recruited, or students can be engaged in the development team.

### Supportive feedback mechanism

In getting rapid and accurate feedbacks from first-hand users, there are three main mechanisms of communication to provide an on-going technical assistance. The first mechanism is via public talks at the hospital under the E-health initiative [48] started by the hospital. Second approach is by engaging faculty members and hospital EMR committee which includes the IT management team in the hospital and finally frequent communication with the breast care nurses who provide direct feedbacks from doctors who use the system on-site. These methods will create understanding among involved parties on how the implementation process is progressing, as well as recognizing strategies to improve the system.

## Phase four: Improving future applications

### Learning from experience

Through this exercise, we have a design plan which is generic to be implemented in other departments in the hospital. It is good that the foundation used in the Breast Cancer Module is the hospital’s existing EMR system, hence we can reuse our upper layer design workflow to match the requirements in the other departments such as Radiology and Pathology. Continuous efforts are under way in maintaining and improving the Surgery, Oncology and Pharmacy modules using the feedback form provided to end users.

The model (Fig. 2) derived from the experience of design and implementation of the module has taught the importance of incorporating a platform for research that has access to both confidential data and editing capabilities, as working on cancer would need identifiers and communication with other bodies.

As aforementioned, the model is in line with the standards laid out by the Clinical Data Interchange Standards Consortium [49] The CDISC standard has also been applied in prominent research on EMR [40, 41, 50].

## Results

### I-Pesakit© Breast Cancer Module outputs

#### General online features

**Fig. 7:**
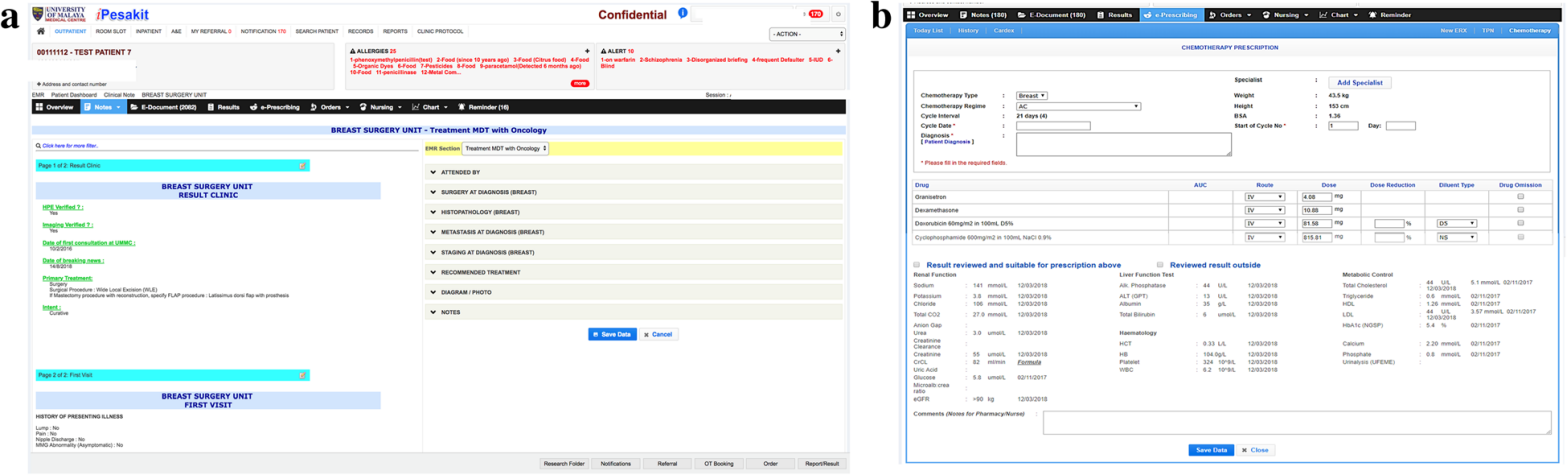
**a** User web interface of the i-Pesakit© Breast Cancer Module in Surgical Unit (**b**) User web interface of the i-Pesakit© Breast Cancer Module in Oncology Department for Chemotherapy e-prescription

The user web interfaces of the i-Pesakit© Breast Cancer Module is demonstrated in Fig. 7a, b. It supports basic and advanced functions that allow multidisciplinary users to experience the interoperability system between clinical departments. Some interesting features of i-Pesakit© Breast Cancer Module include the ability to browse for clinical notes, lab reports, and treatment plans with tabs in current window, specific data editing roles for clinician, nurses and researchers, and data sharing between related clinical departments. More importantly, it implements the point of care data collection method which increases the efficiency of clinical workflow.

#### Audit

**Fig. 8:**
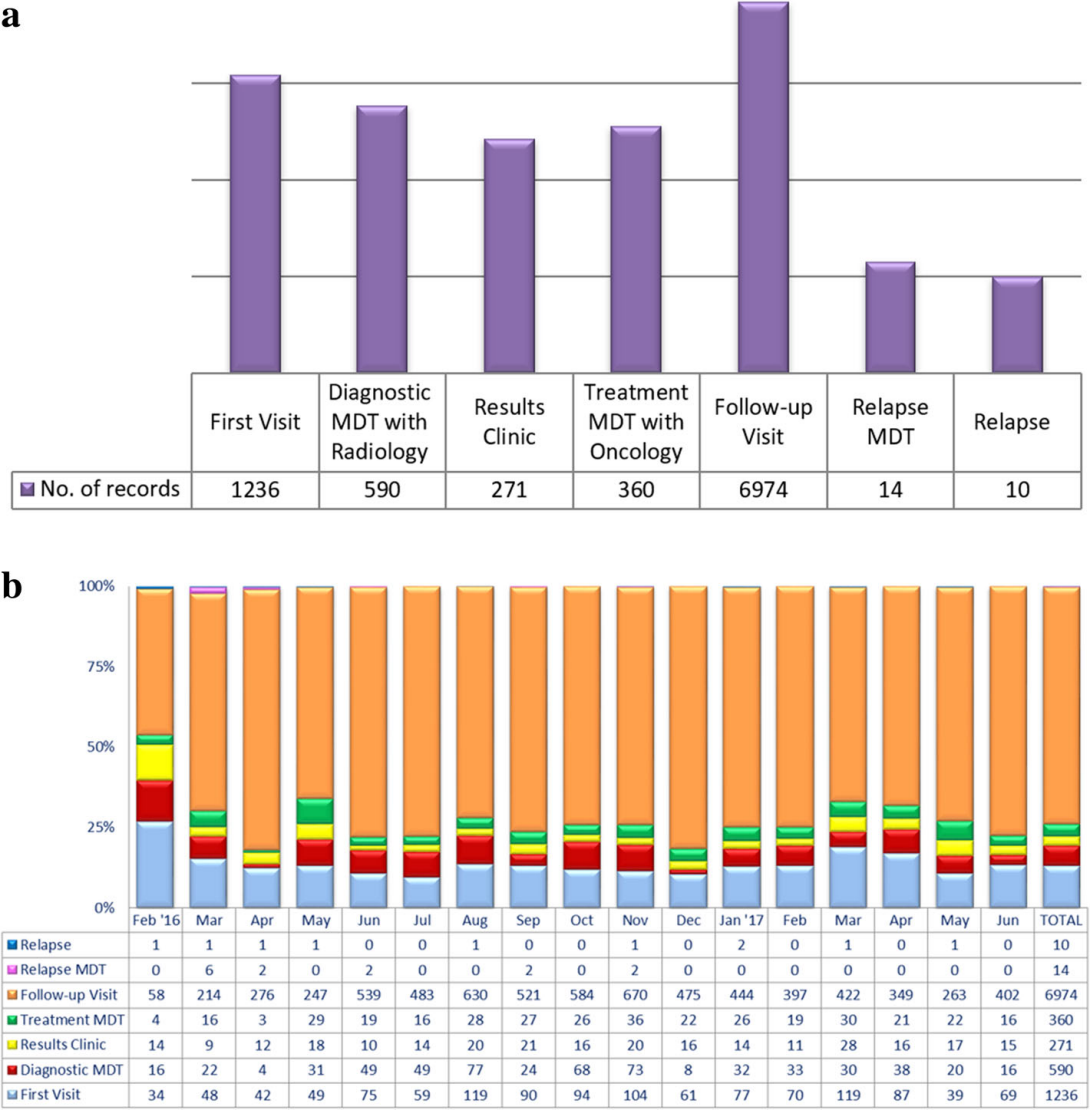
UMMC i-Pesakit© Breast Cancer Module system test and evaluation results (Feb ‘16 - June ’17) (**a**) Summary of UMMC i-Pesakit© Breast Cancer Module usability (**b**) Monthly usage breakdown of UMMC i-Pesakit© Breast Cancer Module in Surgery Department

Clinical audit is important in measuring the hospital’s quality measurement. One of the main output of the i-Pesakit© Breast Cancer Module is its ability to track recurrence. Over the course of 17 months of i-Pesakit© Breast Cancer Module implementation since February 2016, there are a total of 6974 follow-up of cancer cases and 10 recurrences tended by clinicians as shown in Fig. 8a, b. In measuring the hospital performance, it is found that the average duration of a typical breast cancer diagnosis takes 16.8 days. The Treatment MDT meetings of 360 visits would describe the number of new breast cancer patients diagnosed in UMMC. Further work will refine meaningful data capture that will be reported in future works.

## Discussion

The deployment of EMR in hospitals enables electronic reporting and fosters research by establishing affiliations with other institutions and potential external collaborations. In this paper, the i-Pesakit© Breast Cancer Module was developed following the steps in the QIF implementation framework which is a conceptual overview of implementing a healthcare innovation. Phase 1 of the QIF confirmed the need to engage clinicians and real-time workflows. Stakeholder involvement is critical to the success of these implementation efforts. Prior work on implementation of clinical information systems provides broad guidance to inform effective engagement strategies. Phase 2 focuses on the structural features on implementation. In this phase, understanding the multidisciplinary breast cancer clinical workflow is crucial in designing a practical system for users, as well as system enhancement during the testing phase. Phase 3 deals with the support strategies during implementation.

In addition to that, direct engagement with stakeholder is carried out from time to time, in monitoring the system efficiency and receiving feedbacks from users to improve the system’s functionalities. Finally in Phase 4, suggestions are made to improve future application through retrospective system analysis based on feedbacks and suggestions by users. The implementation framework using the QIF framework provides the team a clear path to developing a breast cancer reporting system which incorporates both clinical and research needs for clinical and research organization readiness.

By adopting the QIF framework, we proposed three distinct phases in designing the innovation, which are (i) engagement of stakeholders and following the clinical workflows very closely to ensure usability and accuracy of data captured; (ii) compliance to Personal Data Protection Act (PDPA) and research needs and (iii) de identification of clinical data for research.

The UMMC i-Pesakit© Breast Cancer Module was tested rigorously by the clinicians within the department of surgery. As presented in the Results, in general, clinicians were in favor of the i-Pesakit© Breast Cancer Module system implementation. They believed that the system has improved the quality and clarity of documentation. However, some clinicians described the system as complex and too complicated. They had difficulty at the beginning especially during the familiarization of system flow. While some feel the system, features have increased work efficiency, they also experience some drawbacks such as the point of care data entry resulted in lack of time for eye contact and less clinician-patient communication. This may reduce the effectiveness to create a therapeutic relationship with the patients as clinicians are more focused on the notes in the system as compared to patient consultation. Point of care data entry may not be enough for completeness of data for clinical research [51–53] hence audits to assess completeness of data need to be done. Hiring data managers to obtain complete the data through active engagement with patient and clinicians could be a possible solution Auditing the quality of data is also necessary in maintaining the clinical data integrity in ensuring a certain level of quality [54].

The proposal in this paper is in line with global efforts in digitizing data and clinical workflows [42, 43, 55–57], by paying close attention towards the challenges faced in a middle income country setting.

Many efforts attempting to improve the dire situation of poor records management are being carried out in Malaysia, however, as a developing country, there are some challenges faced which hinders the success. One of the main challenge is lack of funding in supporting medical research costs. In Malaysia, technology is available and has been incorporated into private sectors such as banking and commerce at an accelerated pace. However, as a middle-income country, funding in medical and healthcare focusing on research-based activities is restrained. According to the Malaysian National Budget 2017, budget allocated for medical research is barely USD358m [58], which is less than 10% as compared to other developed countries such as USD380m in Singapore and USD32.3b in the USA [59, 60].

Another challenge faced is the issue of data confidentiality [39]. A governance framework needs to be established to comply with the Personal Data Protection Act 2010 whereby the framework must provide mechanism for data protection of personal information by ensuring secured data sharing in clinical work and research. This includes assuring the system meets the security standards, privacy protection and infrastructure readiness.

Workflow factors that contribute to patient data privacy in accordance to the Personal Data Protection Act 2010 [39] and the National Archives Act 2003 [61] were incorporated early during the planning process to ensure sustainability and compliance through direct engagement with the Medical Records Department. Models of governance is managed by Clinical Investigating Centre (CIC), a committee within the UMMC hospital to address issues brought about by projects like this which is a point of integrated network between clinical workflow and research. The roles of CIC go beyond liaising with Medical Ethics Committee to ensure safe clinical workflow and ethical standards are met. Future work will also include a research module (i-Research) where primary clinical data will be de-identified and used for research-based activities. We propose mirroring of i-Pesakit© to produce the i-Research module as presented in Fig. 3. The i-Research database module provides a carefully controlled research environment for clinicians and scientists to conduct safe and high quality clinical research through the compliance of the Malaysian ICH Good Clinical Practice and is acceptable by the international regulatory authorities. The ability to produce reports on breast cancer outcomes in Malaysia to be used by stakeholders such as clinicians, researchers, and the government is essential for research, hospital performance for policymakers to track outcomes and provide direction in cancer control. This is also a time effective approach in producing new knowledge through systematic data capture design, data mining and analysis, enhancing research and development in the medical and health sciences domain. Future work in incorporating other disciplines into the i-Pesakit© Breast Cancer Module will allow other data sources from oncology, radiology, pathology and pharmacy to be integrated to improve the completion and accuracy of the data reported into the existing audit and research forms. Outcome research in oncology requires accurate clinical and follow-up data. In view of future developments in bioinformatics, research organization readiness to produce accurate clinical and follow-up data is crucial.

## Conclusion

The highlight of the i-Pesakit© Breast Cancer Module presented in this paper is that it was developed in-house with close supervision by clinical and research experts in the hospital using electronic forms and embedded within the EMR system as the opportunity presented during the transitioning between paper based to EMR in UMMC. This multidisciplinary collaboration between Surgery and Oncology departments enhanced clinical workflows and POC data capture, impacts clinical decision making for clinicians, for hospital performance audits and research use. The completion of the UMMC i-Pesakit© Breast Cancer Module would require consolidating multidisciplinary data from various clinical departments. This paper documents the processes and provides a framework for practitioners in the developing world embarking on such endeavor. This framework will potentially guide healthcare entities to prepare for future clinical bioinformatics as technology application in health is still in its infancy in the developing world. Understanding the approach in secondary use of clinical data in the research domain is crucial in data mining in delivering data for meaningful oncology outcomes.

Data mining, building analytical modules and machine learning techniques in prediction works, especially in breast cancer recurrence and survivorship studies are important as they guide national cancer control policy. Direct linkages with other bodies like the National Registry Department and able to report the backend directly to the Malaysian National Cancer Registry will be sought to reduce needed human resources and produce more accurate form of reporting. The accomplishment of the breast cancer module within the EMR will bridge the gap between clinical care and medical informatics research in the future. With additional UMMC biobank facilities and research questions, infrastructure for future bioinformatics research can be conducted.

## Abbreviations

CDISC: Clinical Data Interchange Standards Consortium
CIC: Clinical Investigating Centre
CRF: Case report form
e-CRF: Electronic case report form
EMR: Electronic medical record
HIS: Hospital information system
HL7: Health Level-7
IT: Information technology
MDT: Multidisciplinary team
MYIPO: Intellectual Property Corporation of Malaysia
NEHR: National Electronic Health Records
NPfIT: National Programme for Information Technology
PDPA: Personal Data Protection Act
POC: Point of care
QIF: Quality Implementation Framework
UMMC: University Malaya Medical Centre
